# Ancient tortoise hunting in the southwest Pacific

**DOI:** 10.1038/srep38317

**Published:** 2016-12-06

**Authors:** Stuart Hawkins, Trevor H. Worthy, Stuart Bedford, Matthew Spriggs, Geoffrey Clark, Geoff Irwin, Simon Best, Patrick Kirch

**Affiliations:** 1 Archaeology and Natural History, School of Culture History and Language, College of Asia and the Pacific, The Australian National University, Canberra, ACT 2601, Australia; 2 School of Biological Sciences, Flinders University, Adelaide 5001, SA, Australia; 3 School of Archaeology and Anthropology, College of Arts and Social Sciences, The Australian National University, Canberra, ACT, 2601, Australia; 4 Anthropology, School of Social Sciences, University of Auckland, Private Bag 92019, Auckland 1142, New Zealand; 5 Anthropology, University of California, 232 Kroeber Hall, Berkeley, CA 94720-3710, USA

## Abstract

We report the unprecedented Lapita exploitation and subsequent extinction of large megafauna tortoises (*?Meiolania damelipi*) on tropical islands during the late Holocene over a 281,000 km^2^ region of the southwest Pacific spanning from the Vanuatu archipelago to Viti Levu in Fiji. Zooarchaeological analyses have identified seven early archaeological sites with the remains of this distinctive hornless tortoise, unlike the Gondwanan horned meiolaniid radiation to the southwest. These large tortoise radiations in the Pacific may have contributed to the rapid dispersal of early mobile Neolithic hunters throughout southwest Melanesia and on to western Polynesia. Subsequent rapid extinctions of these terrestrial herbivorous megafauna are likely to have led to significant changes in ecosystems that help explain changes in current archaeological patterns from Post-Lapita contexts in the region.

## Large tortoise distributions and extinctions in the Pacific

The cause of megafauna extinctions during the late Pleistocene to Holocene transition has been a topic of controversy, with either human activities or natural climatic shifts proposed, creating a distinct dichotomy^1^. However, direct evidence for either of these ancient phenomena has been overwhelmingly lacking in most of the tropical Asia-Pacific region^2^. The recent discovery of a novel megafauna species of large tortoise tentatively identified as a meiolaniid, *?Meiolania damelipi*, at the early Teouma cemetery and habitation site on Efate Island in Vanuatu, dated between 3000 and 2500 BP^3^, provides direct evidence for the impact of humans on megafauna in the southwest Pacific. This extinct species, comparable in size to fossil specimens of *Meiolania platyceps* from Lord Howe Island, was identified from large quantities of bones deposited at Teouma during Neolithic human settlement by a maritime culture known as Lapita. Lapita colonists had previously crossed a 350 km water gap from Near Oceania (New Guinea, Bismarck Archipelago, main Solomon Islands chain) into previously uninhabited Remote Oceania (Southeast Solomon Islands, Vanuatu, New Caledonia, Fiji, Tonga, Samoa). At the time of our initial publication, this was the only direct evidence that large-bodied tortoises overlapped with prehistoric human settlement in the Pacific region. Indeed, they were hunted by humans until their extinction, probably accelerated by habitat disturbance and the introduction of invasive biota^3^.

The most likely origin of *?M. damelipi* was believed to have been by flotation from the relict Gondwanan meiolaniid populations in Australia^3^. Such a process had led to the evolution of several species in the Pacific as evidenced by fragmentary fossil finds in the New Caledonian, Loyalty^4^,^5^ and Fijian Islands^6^ and by complete and abundant fossils on Lord Howe Island^7^,^8^. However, dispersal by water north into Vanuatu and Fiji from Gondwanan sources would have been very challenging. This involves the crossing of a significant biogeographic boundary between New Caledonia and Vanuatu, underpinned by strong ocean currents, which sees reptiles floating to New Caledonia from Papuan-Fijian sources, but seldom the other way^9^. Further, Vanuatu, once located closer to Micronesia, has moved counter clockwise north to south to its current position since the Miocene and has fully emerged only in the last 2 million years^10^; and so an Indo-Pacific or neo-tropical origin must also be considered. *?Meiolania damelipi* skull bones, including robust and highly diagnostic parietal horns that typify this genus, were not identified at Teouma^3^. Horns dominate the *M. mackayi* remains on Walpole Island in New Caledonia^4^ and are common for *M. platyceps* at Lord Howe Island^7^. Therefore, the referral of the Vanuatu tortoise to *Meiolania* was tentative and its origins and region of radiation currently remain uncertain as does the extent of early prehistoric tortoise hunting in Oceania. Since then Sterli^11^ has argued, based on the robusticity of long bone characteristics described by White *et al*.^3^, that *?M. damelpi* cannot in fact belong to the Meiolaniidae. Ongoing osteological and ancient DNA studies may resolve these uncertainties in its true affinities.

**Figure 1:**
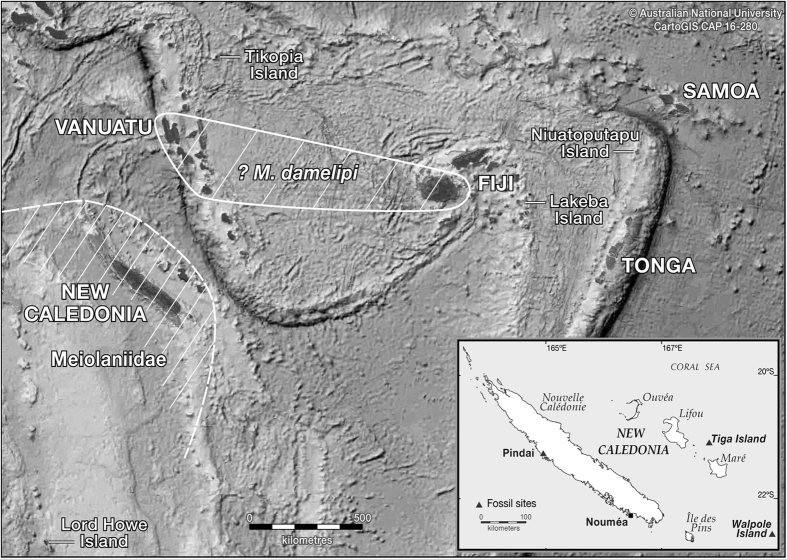
The Southwest Pacific Region showing the current distribution of two distinctive land turtle types the hornless *?Meiolania damelipi* from archaeological sites in Vanuatu and Fiji and horned Meiolaniidae further south from fossil sites in New Caledonia and on Lord Howe Island. Archaeological sites which were checked but did not contain tortoise bones from Tikopia, Niuatoputapu and Lakeba Islands are also shown. ANU, CartoGIS CAP 16–280. Adobe Illustrator version Creative Cloud (CC). http://www.adobe.com/au/products/illustrator.html.

**Figure 2:**
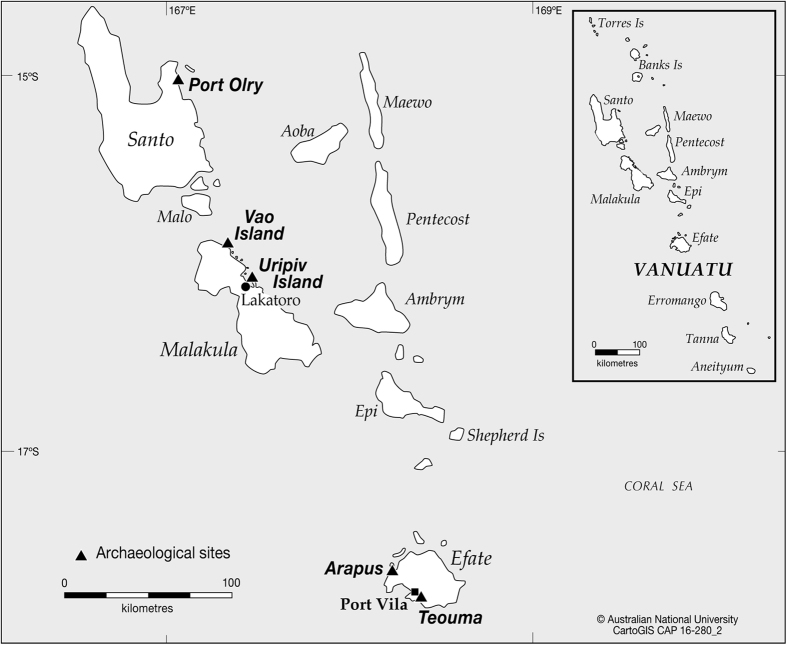
Vanuatu, showing the locations of five archaeological sites with *?M damelipi* type tortoise bones. ANU, CartoGIS CAP 16-280-2. Adobe Illustrator version Creative Cloud (CC). http://www.adobe.com/au/products/illustrator.html.

**Figure 3:**
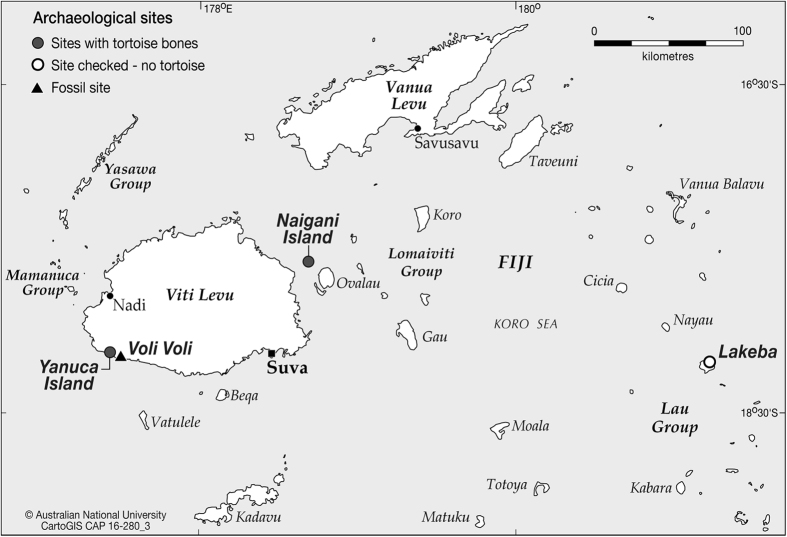
Fiji, showing the locations of archaeological sites on Naigani and Yanuca islands, the fossil site of Voli Voli and Lakeba Island. ANU, CartoGIS CAP 16-280-3. Adobe Illustrator version Creative Cloud (CC). http://www.adobe.com/au/products/illustrator.html.

Here we report continuing research on new and previously excavated archaeofaunal assemblages and the discovery of a much wider extent of large-bodied tortoise hunting in the Pacific than was previously known. Seven early colonising archaeological assemblages containing tortoise bones from Vanuatu and Fiji have now been identified (Figs 1, 2 and 3, Supplementary Figs S1–S3; Supplementary Tables S1–S12, SI Text). These sites encompass an Oceanic region of tortoise hunting of approximately 281,000 km^2^ from northern Santo to central Efate in Vanuatu, to Viti Levu and Naigani Island in Fiji (Figs 1, 2 and 3). Such a widespread region of prehistoric large tortoise hunting is unparalleled in the global archaeological record. This area is likely to extend further southwest into New Caledonia, given meiolaniid fossil presence on Tiga and Wapole Islands (Fig. 1), and unsubstantiated reports of meiolaniid bones in Lapita sites on the south coast of the Grand Terre mainland^5^ that have yet to be published in any detail.

## Results

The new Vanuatu sites include abundant tortoise bones at the Lapita site on Vao Island (Supplementary Table S3) and one bone at the Late Lapita site on Uripiv Island (Supplementary Table S5), located off the northeast coast of Malakula (SI Text). Tortoise bones are also now known from the Port Olry Lapita site (Supplementary Table S4) in the northeast of Santo Island (SI Text) and from the immediately Post-Lapita Arapus site (Supplementary Table S6) on Efate Island in central Vanuatu. Many of the bones appear burnt, with cut marks and butchery fractures (Supplementary Figs S4 and S5, Supplementary Table S10), and all tortoise remains are associated with early human settlement of Vanuatu between 3000-2700 BP (SI Text, Supplementary Table S1). These now include dentaries and maxillae (Supplementary Table S9) with the remaining, less dense, part of the skull likely fragmented during butchery, cooking, trampling and post-depositional breakage.

The exceptionally dense and highly characteristic meiolaniid horns, found in Australian, Lord Howe Island, and New Caledonian sites, remain absent from Vanuatu assemblages, indicating that the Vanuatu tortoise was not horned. To the east, we also report the first cases of tortoise bones from Fijian Lapita sites (Fig. 3). These include only single post-cranial bones from Yanuca off Viti Levu and Naigani just off the northeast coast of that island (SI Text). However, it must be noted that Fijian Lapita sites are generally poorly preserved and bereft of abundant terrestrial vertebrate remains^12^. Our examinations show that these bones, which were previously misidentified in the case of Yanuca (Supplementary Fig. S2, Supplementary Table S7) and remained unidentified at Naigani (Supplementary Fig. S1, Supplementary Table S8), share a greater morphological similarity with the Vanuatu tortoise *?Meiolania damelipi* than with *Meiolania platyceps* (Supplementary Figs S1–S3).

Turning to other adjacent island groups, Tikopia in the southeast Solomon Islands, Lakeba in eastern Fiji, and Niuatoputapu in northern Tonga, each have well reported vertebrate fauna^13^, and recent reanalysis of these collections proved negative for tortoise remains, which are distinctive compared to those of similar-sized sea turtles^3^.

## Discussion and Conclusions

The archaeological evidence supports long-distance voyaging by Lapita sailors^14^,^15^,^16^ as well as mobile harvesting of these large sedate herbivorous tortoises (ref. 17, SI Text, Supplementary Table S14). In a virtual replay of prehistory, Historic-period European long distance mariners exploited dense abundances of giant tortoises for provisions in the Indian Ocean and the Galapagos, literally capturing thousands, with extirpation occurring on several islands about one or two centuries after first human arrival^18^,^19^.

Previously considered ‘push’ factors including agricultural limits, population growth, resource depression and even malaria have been touted as the main drivers for episodic Lapita dispersal^20^. Exploitation of initially abundant native fauna (marine fish and shellfish, large flightless birds, land crocodilians, giant iguanas, fruit bats) as subsistence resources was also crucially important as a ‘pull’ factor wherever Lapita groups settled in Remote Oceania^13^. However, our new discoveries indicate that ‘pull’ factors for early migratory and highly mobile Lapita hunters were perhaps much more significant than previously thought for island groups that had extant tortoise populations. The predicted dense populations and high biomass of large tortoises based on populations situated on predator-free tropical islands elsewhere^21^,^22^, combined with their ease of hunting would have presented them as optimal resources. The Ideal Free Distribution Model predicts that Lapita voyagers would initially be free to move to the most productive habitats and that productivity would decline with increasing population density and resource intensification^20^. This may have contributed to increased rates of Neolithic dispersal and colonisation as far as western Polynesia during the late Holocene once exploratory parties had moved beyond the previously-occupied main Solomon Islands chain.

The mobility of prehistoric tortoise hunters is likely to have resulted in an increasingly expanding tortoise depletion zone as Lapita people quickly moved to un-depleted resource patches in other island regions. Rapid extinction of these keystone herbivores followed by ecological fragmentation is predicted based on giant tortoise extinctions on other tropical islands^23^ and observed in the archaeological and historical record in the Indian Ocean and Galapagos Islands^22^. This would have resulted in dramatic changes in Pacific Island palaeoecology as well as presenting significant consequences for post-colonisation human settlement and society. The widespread extinction of tortoises and reductions in other native fauna in the Vanuatu-Fiji region appear to have coincided with a subsequent period of reduced mobility as ‘pull’ factors became less attractive, culminating in the transition of Lapita into many distinctive Post- Lapita regional island cultures in Remote Oceania^24^.

More work is required to illuminate the full extent of *?M. damelipi* distribution and ancient human hunting in the Pacific. We predict that the re-analysis of existing Pacific vertebrate collections that sample the first phase of human impacts will be productive, as will the discovery and excavation of new Lapita sites around the periphery of the Vanuatu-Fiji region. This is an urgent objective before encroaching coastal development, climate change and associated sea level rise begin to increasingly impact on the archaeological record.

## Materials and Methods

This study was conducted to discover direct evidence of the extent of large-bodied tortoise distribution and hunting in the southwest Pacific region of Remote Oceania during early Neolithic settlement in the Late Holocene. For this purpose, ten turtle vertebrate assemblages from early colonising archaeological sites in the region were analysed or reanalysed with special attention paid to the systematic palaeontology of vertebrate specimens, morphometrical analysis, quantification of tortoise skeletal elements as well as bone modifications that could be ascribed to tortoise processing by prehistoric humans during butchery and consumption.

Identification of large tortoise bones from five Lapita sites in Vanuatu (Teouma, Vao, Uripiv, Arapus, Port Olry) and two Lapita sites in Fiji (Yanuca, Naigani) were made using comparisons with type and other specimens of *Meiolania platyceps* from Lord Howe Island in the Australian Museum in Sydney and *?Meiolania damelipi* from Teouma. The Teouma material of *?Meiolania damelipi* included that identified by White *et al*.^3^, the type specimens of which are in the Australian Museum, and other specimens identified by one of the authors S.H. (numbers with AW prefix or SCH prefix) that are currently held at the Australian National University in Canberra. Identification of the new tortoise specimens was made by direct comparison with reference to key morphological land marks and features following Gaffney^8^ and for *?M. damelipi* to those identified by White *et al*.^3^. The specimens were identified for each provenance unit to skeletal element, side and portion based on diagnostic features as well as zones (Supplementary Table S13). Minimum Number of Elements (MNE) and Minimum Number of Individuals (MNI) were calculated by counting the most frequent side and portion of each skeletal element by manual overlap calculations for the entire assemblage. Most turtle shell fragments were excluded due to high rates of fragmentation.

Bone modifications were observed at the macroscopic level and by hand lens (x10) followed up by binocular microscope (Zeiss Stemi 2000-C W-PI, 10x/23 magnification) for closer inspection of potential surface features. The presence of cut marks, with no distinction made between V- shaped lines, striations, or shoulder stria, were identified by general anatomical zone (e.g. proximal, shaft, distal). Here we only include the proportions of skeletal elements with cut marks present (Supplementary Table S10) as this is considered to make the fewest assumptions given the variability in inherent bone characteristics between skeletal elements and different portions of skeletal elements^25^,^26^. Evidence for burning was based on discoloration using scores (0- tan/cream, 1- < half black, 2- > half black, 3- fully black, 4- < half grey/white/blue, 5- > half grey/white/blue, 6- fully grey/white/blue)^27^. Discoloration was cross referenced by observing textual traits such as bone surface cracking, flaking, exfoliation and shrinking^27^,^28^. However, potential differences in the inorganic content of large reptile bones compared with large mammal specimens conducted in other studies^27^,^28^ and the potential bias for temperature inferences meant that we only included presence/absence of burning (Supplementary Table S10) to infer cooking or burning bone for fuel. Nonetheless we note most of the bones were stage 0 with discoloured bones appearing as stages 1–2. The ubiquitous presence of fireplaces, charcoal and fire cracked rocks and other midden remains throughout the provenance units at these open beach sites provide additional proxies for fire based activities. The presence/absence of fresh regular bone fractures with smooth fracture surfaces, or percussion scars and pits associated with Lapita butchery practices, were identified and recorded for each specimen. These taphonomic data are summarised by element in Supplementary Table S10. Isotope and direct C14 data for tortoise skeletal elements from Teouma have been reported elsewhere^3^,^17^. In addition, the Yanuca femur (#16/81/2/3/4/1) was sent for isotopic analysis and C14 dating at the University of Oxford. While isotopic data extraction was successful (Supplementary Table S14), there was not enough collagen for radiocarbon determination and the femur has been destroyed except for a sample of powdered bone.

## Additional Information

**How to cite this article**: Hawkins, S. *et al*. Ancient tortoise hunting in the southwest Pacific. *Sci. Rep.* **6**, 38317; doi: 10.1038/srep38317 (2016).

**Publisher's note:** Springer Nature remains neutral with regard to jurisdictional claims in published maps and institutional affiliations.

## Supplementary Material

Supplementary Information

Supplementary Table S2

Supplementary Table S3

Supplementary Table S13

## References

[b1] Johnson C. N. . What caused extinction of the Pleistocene megafauna of Sahul? Proc. R. Soc. B. 283, 20152399 (2016). 10.1098/rspb.2015.2399 PMC4760161 26865301

[b2] Corlett R. T. Megafaunal extinctions and their consequences in the tropical Indo-Pacific In Altered Ecologies: Fire, Climate and Human Influence on Terrestrial Landscapes, Canberra (eds Haberle S., Stevenson J., Prebble M.) 117–131 (Terra Australis, 2010).

[b3] White A., Worthy T., Hawkins S., Bedford S. & Spriggs M. Megafaunal meiolaniid horned turtles survived until early human settlement in Vanuatu, Southwest Pacific. PNAS. 107(35), 15512–15516 (2010). 20713711 10.1073/pnas.1005780107 PMC2932593

[b4] Gaffney E. S., Balouet J. C. & Broin F. D. New occurrences of extinct meiolaniid turtles in New Caledonia. American Museum Novitates. 2800, 1–6 (1984).

[b5] Balouet J. C. The fossil vertebrate record of New Caledonia In Vertebrate Palaeontology of Australasia (eds Vickers P., Monaghan J. M., Bajrd R. F. & Rich T. H.) 1381–1409 (Monash University Publications, 1991).

[b6] Worthy T., Anderson A. & Molnar R. Megafaunal expression in a land without mammals - the first fossil faunas from terrestrial deposits in Fiji (Vertebrata: Amphibia, Reptilia, Aves). Senckenbergiana biologica. 79, 237–242 (1999).

[b7] Gaffney E. S. Cranial morphology of the extinct horned turtle, *Meiolania platyceps*, from the Pleistocene of Lord Howe Island. Bulletin of the American Museum of Natural History. 175(4), 361–480 (1983).

[b8] Gaffney E. S. The postcranial morphology of *Meiolania platyceps* and a review of the Meiolaniidae. Bulletin of the American Museum of Natural History. 229, 1–166 (1996).

[b9] Hamilton A. M., Klein E. R. & Austin C. C. Biogeographic Breaks in Vanuatu, a Nascent Oceanic Archipelago. Pacific Science. 64(2), 149–159 (2010).

[b10] Gibbons J. R. H. The biogeography and evolution of Pacific island reptiles and amphibians In Biology of Australasian frogs and reptiles (eds Grigg G., Shine R., Ehmann H.) 125–142 (Royal Zoological Society of New South Wales, 1985).

[b11] Sterli J. A review of the fossil record of Gondwanan turtles of the clade Meiolaniformes. Bulletin of the Peabody Museum of Natural History. 56(1), 21–45 (2015).

[b12] Worthy T. & Clark G. Bird, mammal and reptile remains In The early prehistory of Fiji (eds Anderson A., Clark G.) 231–258 (Terra Australis, 2009).

[b13] Kirch P. V. On the Road of the Winds: An archaeological history of the Pacific islands before European contact. (University of California Press, 2000).

[b14] Dickinson W., Bedford S. & Spriggs M. Petrography of temper sands in 112 reconstructed Lapita pottery vessels from Teouma (Efate): archaeological implications and relations to other Vanuatu tempers. Journal of Pacific Archaeology. 4(2), 1–20 (2013).

[b15] Galipaud J. C., Reepmeyer C., Torrence R., Kelloway S. & White P. Long‐distance connections in Vanuatu: New obsidian characterisations for the Makué site, Aore Island. Archaeology in Oceania. 49(2), 110–116 (2014).

[b16] Irwin G. & Flay R. G. Pacific colonisation and canoe performance: Experiments in the science of sailing. Journal of the Polynesian Society. 124(4), 419–443 (2015).

[b17] Kinaston R. . Lapita diet, subsistence strategies and methods of animal husbandry in Remote Oceania: new stable isotope evidence from the 3000-year-old Teouma site, Efate Island, Vanuatu. PLOS ONE. 9(3), e90376 (2014). 24598939 10.1371/journal.pone.0090376 PMC3944017

[b18] van den Burgh J. Expedition of the California Academy of Sciences to the Galapagos Islands 1905–1906. Proc. Cal. Acad. Sc. 2, 203–374 (1914).

[b19] Cheke A. & Hume J. Lost land of the Dodo: The Ecological History of Mauritius, Réunion, and Rodrigues (T & A.D. Poyser, 2008).

[b20] Kennett D. J., Anderson A. & Winterhalder B. The ideal free distribution, food production, and the colonization of Oceania In Behavioral ecology and the transition to agriculture (eds Kennett D., Winterhalder B.) 265–288 (University of California Press, 2006).

[b21] Coe M. J., Bourn D. & Swingland I. R. The biomass, production and carrying capacity of giant tortoises on Aldabra. Philosophical Transactions of the Royal Society B: Biological Sciences. 286(1011), 163–176 (1979).

[b22] Rhodin A. G. J. . Turtles and tortoises of the world during the rise and global spread of humanity: First checklist and review of extinct Pleistocene and Holocene Chelonians. IUCN, Chelonian Research Monographs. 5, doi: 10.3854/crm.5.000e (2015).

[b23] Hansen D. M. & Galetti M. The forgotten megafauna. Science. 324(5923), 42–43 (2009). 19342573 10.1126/science.1172393

[b24] Valentin F., Herrscher E., Bedford S., Spriggs M. & Buckley H. Evidence for social and cultural change in Central Vanuatu during the first millennium BC: comparing funerary and dietary patterns of the first and later generations at Teouma, Efate. Journal of Island and Coastal Archaeology. 9(3), 381–399 (2014).

[b25] Egeland C. P. Carcass processing intensity and cutmark creation: an experimental approach. The Plains Anthropologist. 48(184), 39–51 (2003).

[b26] Lyman R. L. Analyzing cut marks: lessons from artiodactyl remains in the northwestern United States. Journal of Archaeological Science. 32(12), 1722–1732 (2005).

[b27] Stiner M. C., Kuhn S. L., Weiner S. & Bar-Yosef O. Differential burning, recrystallization, and fragmentation of archaeological bone. Journal of Archaeological Science. 22(2), 223–237 (1995).

[b28] Shipman P., Foster G. & Schoeninger M. Burnt bones and teeth: an experimental study of color, morphology, crystal structure and shrinkage. Journal of archaeological science. 11(4), 307–325 (1984).

