# Supplementary material for: Ancient tortoise hunting in the southwest Pacific
Source: Sci Rep. 2016 Dec 6;6:38317. doi: 10.1038/srep38317 (PMC5138842; doi:10.1038/srep38317)
Supplement: Supplementary Information [file srep38317-s1.pdf]

Supplementary Materials for Ancient tortoise hunting in the southwest Pacific

Stuart Hawkins, Trevor H. Worthy, Stuart Bedford, Matthew Spriggs, Geoff Clark, Geoff Irwin, Simon Best and Patrick Kirch

Correspondence to: stuart.hawkins@anu.edu.au

This file includes:

Supplementary Text

Figs. S1 to S5

Tables S1 to S14

SI Figures

Fig. S1: *?Meiolania damelipi*. Left humeri; 1: dorsal view, 2: anterior view, 3: posterior view, 4: ventral view. A. Teouma (#SCH: 589); B. Vao (#SCH: 251); C. Port Olry (#SCH: 14); D. Naigani (#SCH: 150). Abbreviations: ect f (ectepicondylar foramen), int fos (intertubercular fossa), lat (lateral process), med (medial process), tro (trochlea), dp fos (dorsal proximal shaft fossa), vp fos (ventral proximal shaft fossa), ap ma (anterior proximal shaft muscle attachment), pp ma (posterior proximal shaft muscle attachment). Scale = 5 cm.

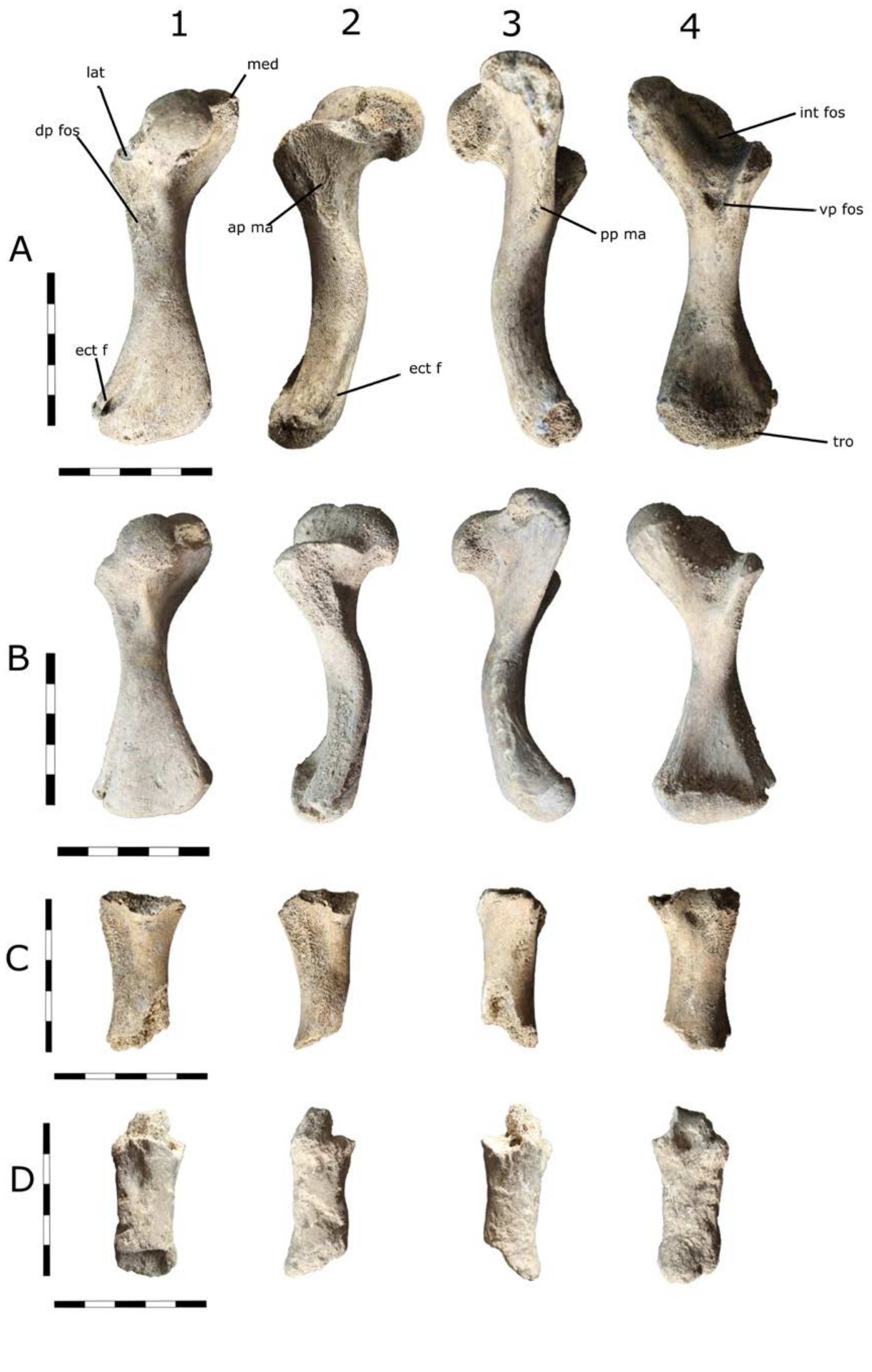
1
2
3
4
A
B
C
D
lat
med
dp fos
ap ma
pp ma
int fos
vp fos
ect f
ect f
tro

Fig. S2: *?Meiolania damelipi*. Left femora; 1: dorsal view, 2: posterior view, 3: anterior view, 4: ventral view. A. Teouma (#AW 404); B. Vao (#SCH 54); C Uripiv (#SCH 250); D. Yanuca (#16/81/2/3/4/1). Abbreviations: fib con (fibular condyle), int fos (intertrochanteric fossa), t maj (trochanter major), t min (trochanter minor), tib con (tibia condyle), ap ma (anterior proximal shaft muscle attachment scar), pp ma (posterior proximal shaft muscle attachment scar). Scale = 5 cm.

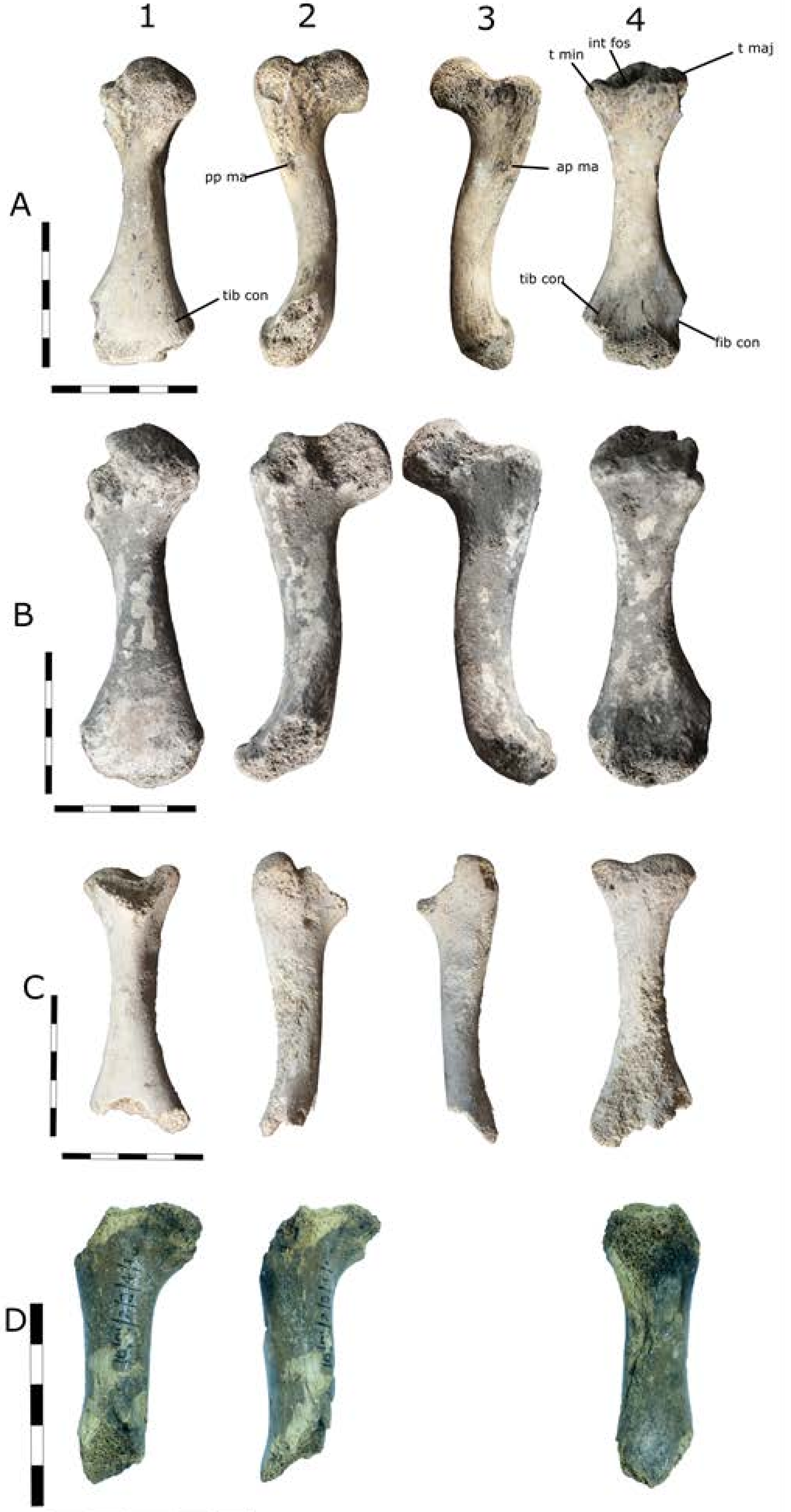
1
2
3
4
A
B
C
D
t min
int fos
t maj
pp ma
ap ma
tib con
tib con
fib con

Fig. S3: *?Meiolania damelipi*. Left scapulae (acromion/dorsal scapular process/glenoid); 1: lateral view, 2: medial view. A. Teouma (#AW: 167), B. Vao (#SCH: 91), C. Arapus (#SCH 267). Abbreviations: ac (acromion process), ac l (acromion lateral muscle attachment), ca (coracoid articulation), gl (glenoid), dsp (dorsal scapular process). Scale = 5 cm.

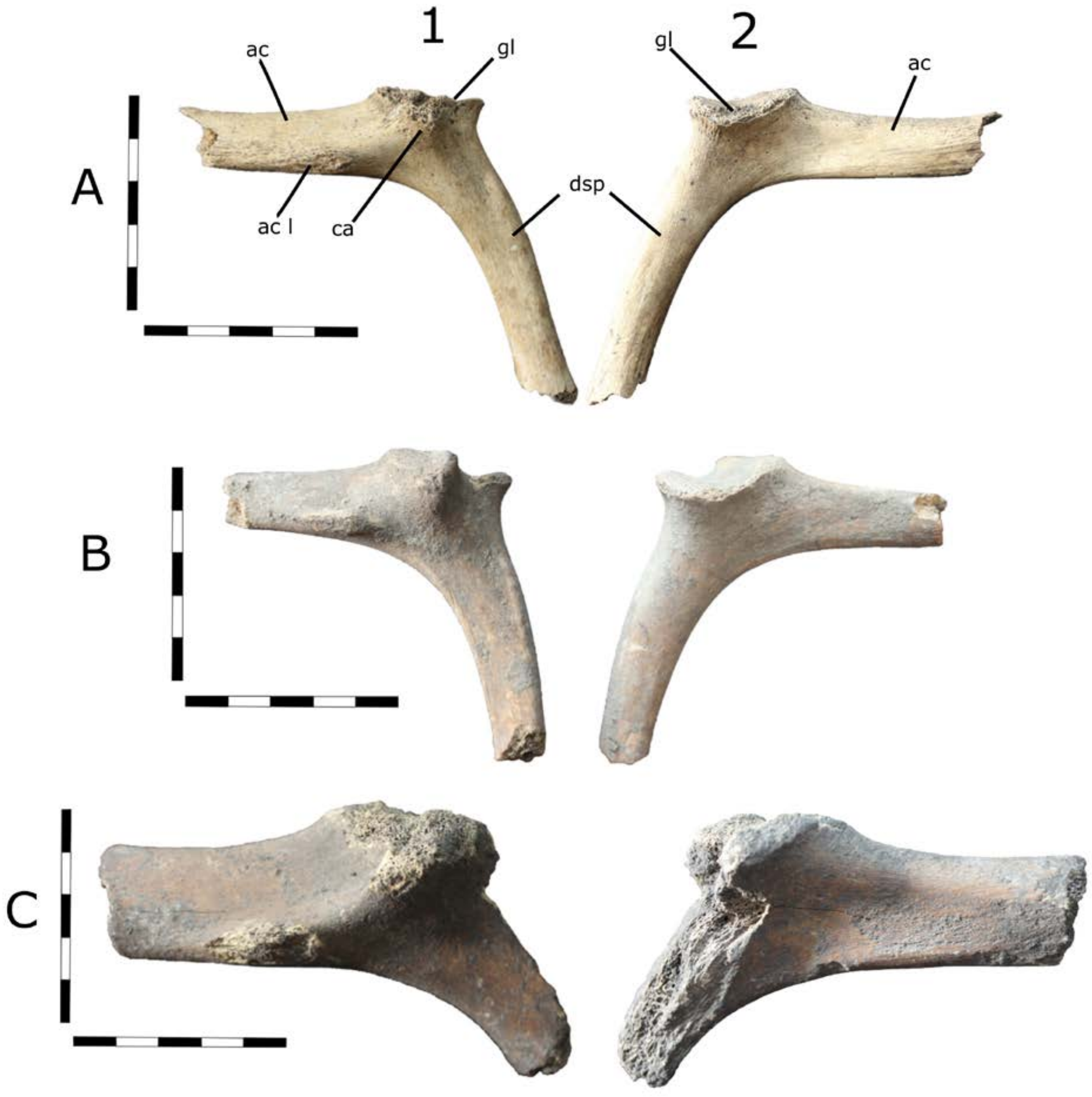

Fig. S4: *?Meiolania damelipi*, left femur (Teouma #SCH 853, 22/06/2009), burnt stage 1 (less than half the bone is black), and transverse regular fracture with percussion pit and scar; evidence of butchery and cooking practices. Scale = 5 cm.

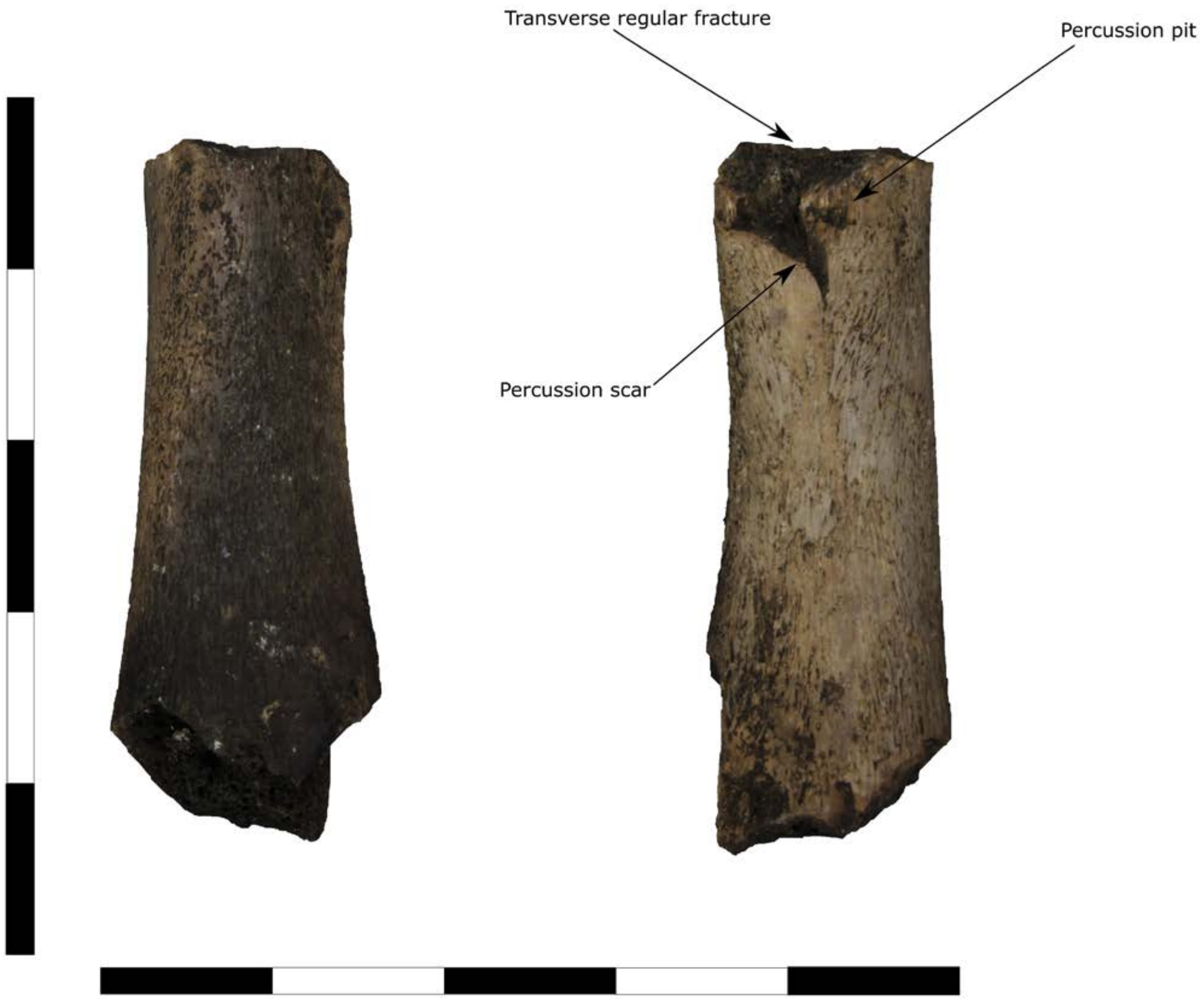

Fig. S5: *?Meiolania damelipi*, left humerus (Teouma #AW 81, 20/07/2004) with 18 parallel cut marks complete with shoulder effect stria parallel to v-shaped grooves along the proximal lateral shaft from cutting with stone artefacts during flesh removal.

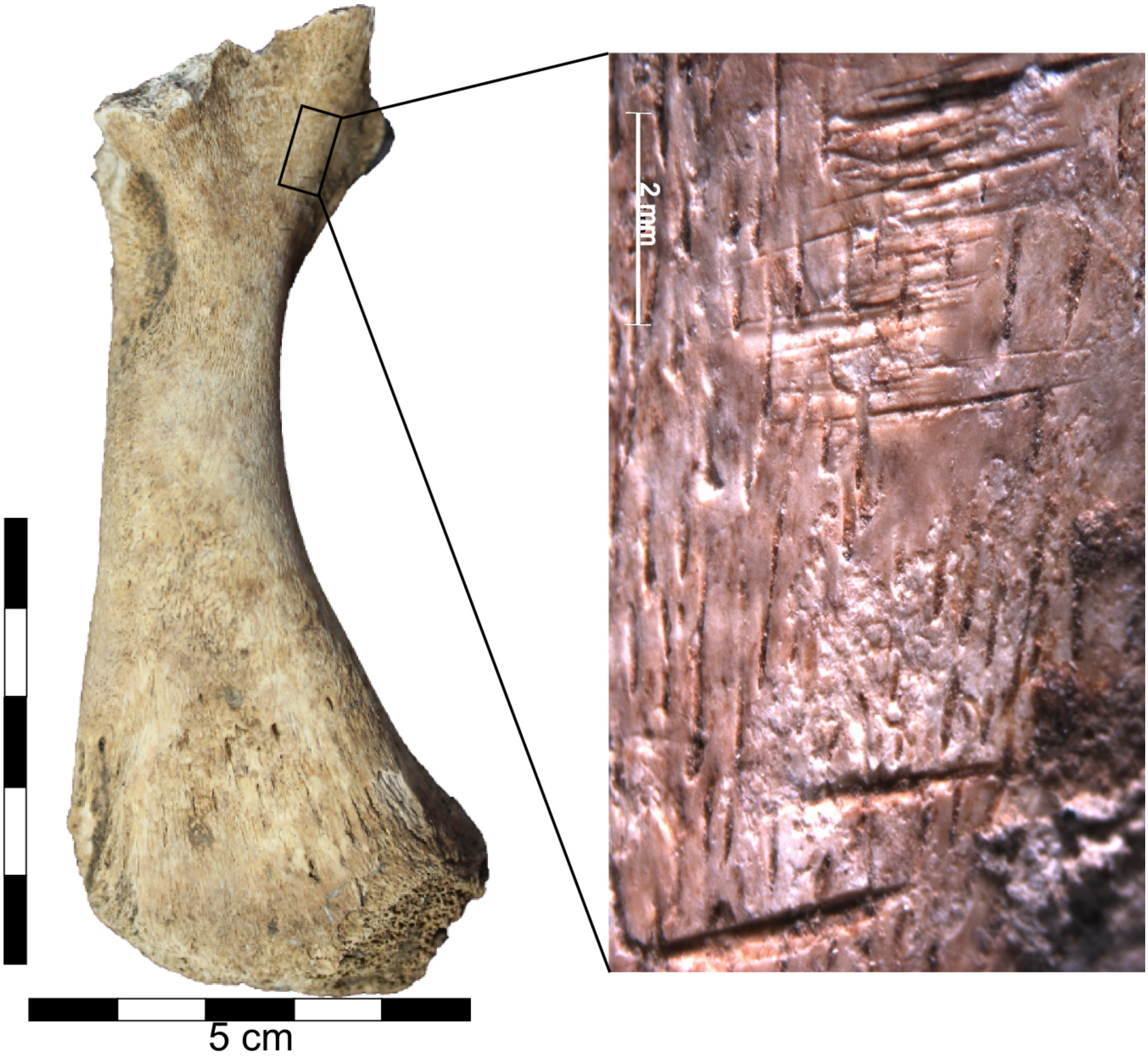

SI Tables

Table S1: List of Pacific archaeological sites with identified *?Meiolania damelipi* tortoise remains.

Site	Island	Country	Ceramic period associated with tortoise bones	Age	Site Type	Excavation size m²	NISP	Recovery method
Teouma	Efate	Vanuatu	Lapita-Erueti	3000-2500 BP	Coastal Settlement/cemetery	473	1915	1-2mm wet and 5mm dry
Arapus	Efate	Vanuatu	Arapus	2900-2700	Coastal settlement	70	2	1-2mm wet and 5mm dry
Vao	Vao	Vanuatu	Lapita	3000-2600	Coastal Settlement/cemetery	36	1672	1-2mm wet and 5mm dry
Vilavi	Uripiv	Vanuatu	Lapita	2950-2600	Coastal Settlement/cemetery	75	1	1-2mm wet and 5mm dry
Port Olry	Santo	Vanuatu	Lapita	2900-2700	Coastal settlement	10	5	1-2mm wet and 5mm dry
VL 21/5	Naigani	Fiji	Lapita	3000-2410 BP	Coastal settlement	120	1	7, 6.5, 3.5, 3, 2.5mm dry
VL 16/81	Yanuca	Fiji	Lapita	2930-2730 BP	Coastal rockshelter settlement	47	1	not screened

Table S2: Teouma primary data for *?Meiolania damelipi* identifications to skeletal element by side and portion. In column 1: Year of excavation; column 2: catalogue number AMF (Australian Museum Palaeontology Department), or AW (Arthur White), SCH (Stuart Hawkins) not prefixed; column 3: the field sample number often with multiple specimens; column 4: Excavation area; column 5: Excavation square; column 6: feature number; column 7: sedimentary layer; column 8: arbitrary within-layer excavation spit usually 10 or 20 cm; column 9: depth from excavation surface; column 10: EL=skeletal element; column 11: side; column 12: skeletal portion; column 13: NISP= Number of Identified Specimens; column 14: specimen weight (gm).

Table S3: Vao primary data for *?Meiolania damelipi* identifications to skeletal element by side and portion. In column 1: Year of excavation; column 2: catalogue number SCH (Stuart Hawkins) not prefixed; column 3: the field sample number often with multiple specimens; column 4: Excavation area; column 5: Excavation square; column 6: feature number; column 7: sedimentary layer; column 8: arbitrary within-layer excavation spit usually 10 or 20 cm; column 9: depth from excavation surface; column 10: EL=skeletal element; column 11: side; column 12: skeletal portion; column 13: NISP= Number of Identified Specimens; column 14: specimen weight (gm).

Table S4: Port Olry primary data for *?Meiolania damelipi* identifications to skeletal element by side and portion. In column 1: Year of excavation; column 2: catalogue number SCH (Stuart Hawkins) not prefixed; column 3: the field sample number often with multiple specimens; column 4: Excavation area; column 5: Excavation square; column 6: feature number; column 7: sedimentary layer; column 8: arbitrary within-layer excavation spit usually 10 or 20 cm; column 9: depth from excavation surface; column 10: EL=skeletal element; column 11: side; column 12: skeletal portion; column 13: NISP= Number of Identified Specimens; column 14: specimen weight (gm).

Season	ID	FS	Area	Sq	Feature	Layer	Spit	Depth	EL	SD	POR	NISP	Weight gm
2011	12	131	1	TP8				40-60	CAD	r	prs co	1	2.69
2011	13	136	1	TP8				60-75	PV	l	pb md co	1	0.76
2011	14	36	1	TP9				40-60	HM	l	shpr1/2	1	9.88
2011	15	26	1	TP9				0-20	CRN	r	mx co	1	2.79
2011	18	32	1	TP9				20-40	CE		pr ap	1	8.7
												5	24.82

Table S5: Uripiv primary data for *?Meiolania damelipi* identifications to skeletal element by side and portion. In column 1: Date of recovery; column 2: catalogue number SCH (Stuart Hawkins) not prefixed; column 3: the field sample number often with multiple specimens; column 4: Excavation area; column 5: Excavation square; column 6: sedimentary layer; column 7: depth from excavation surface; column 8: EL=skeletal element; column 9: side; column 10: skeletal portion; column 11: NISP= Number of Identified Specimens; column 12: specimen weight (gm).

Date	ID	FS	Area	Sq	Layer	Depth	EL	SD	POR	NISP	Weight gm
8/18/2001	250	878		TP8	6	220-240cm	FM	r	psh co	1	22.87

Table S6: Arapus primary data for *?Meiolania damelipi* identifications to skeletal element by side and portion. In column 1: Date of recovery; column 2: catalogue number SCH (Stuart Hawkins) not prefixed; column 3: the field sample number often with multiple specimens; column 4: Excavation square; column 5: sedimentary layer; column 6: depth from excavation surface; column 7: EL=skeletal element; column 8: side; column 9: skeletal portion; column 10: NISP= Number of Identified Specimens; column 11: specimen weight (gm).

Date	ID	FS	Sq	Layer	Depth	EL	SD	POR	NISP	Weight gm
6/24/1999	311	3101V	TP28		200-210	PV	r	pb md bdr	1	5.81
6/22/1999	267	4093F	TP4		135-150	PEC	l	arc + sc pr	1	49.37
Total									2	55.18

Table S7: Yanuca primary data for *?Meiolania damelipi* identifications to skeletal element by side and portion. In column 1: Date of recovery; column 2: catalogue number; column 3: Excavation area; column 4: Excavation square; column 5: ceramic zone; column 6: excavation spit; column 7: depth from excavation surface; column 8: EL=skeletal element; column 9: side; column 10: skeletal portion; column 11: NISP= Number of Identified Specimens; column 12: specimen weight (gm).

Date	ID	Area	Sq	Zone	Spit	Depth	EL	SD	POR	NISP	Weight gm
1965-66	16/81/2/3/4/1		Trench 2	3	4	165 cm	FM	r	psh co	1	n/a

Table S8: Naigani primary data for *?Meiolania damelipi* identifications to skeletal element by side and portion. In column 1: Date of recovery; column 2: catalogue number SCH (Stuart Hawkins) not prefixed; column 3: Excavation area; column 4: Excavation square; column 5: sedimentary layer; column 6: depth from excavation surface; column 7: EL=skeletal element; column 8: side; column 9: skeletal portion; column 10: NISP= Number of Identified Specimens; column 11: specimen weight (gm).

Date	ID	Area	Sq	Layer	Depth	EL	SD	POR	NISP	Weight gm
1981	150		14	A3		HM	l	shpr1/2 co	1	9.09

Table S9: Identified tortoise skeletal elements by NISP and MNE for the seven Lapita sites from Vanuatu (Teouma, Vao, Uripiv, Port Olry, Arapus) and Fiji (Yanuca, Naigani). Teouma MNI = 68, Vao MNI = 49, Port Olry, Uripiv, Arapus, Yanuca, Naigani MNI = 1. Large numbers of carapace and plastron fragments were not included.

Skeletal elements	Teouma		Vao		Uripiv		Arapus		Port Olry		Naigani		Yanuca	
	NISP	MNE	NISP	MNE	NISP	MNE	NISP	MNE	NISP	MNE	NISP	MNE	NISP	MNE
Maxillae	15	5	35	16	0	0	0	0	1	1	0	0	0	0
Mandibles	9	6	46	13	0	0	0	0	0	0	0	0	0	0
Unidentified tooth row fragment	0	0	1	0	0	0	0	0	0	0	0	0	0	0
Hyoids	1	1	1	1	0	0	0	0	0	0	0	0	0	0
Cervical vertebra	52	13	131	43	0	0	0	0	1	1	0	0	0	0
Thoracic vertebra	3	1	22	16	0	0	0	0	0	0	0	0	0	0
Caudal vertebra	31	25	69	47	0	0	0	0	0	0	0	0	0	0
Vertebra	9	0	8	0	0	0	0	0	0	0	0	0	0	0
Dermal shell fragments	21	1	1	1	0	0	0	0	0	0	0	0	0	0
Ribs	1	1	2	2	0	0	0	0	0	0	0	0	0	0
Pectorals (dorsal, acromion, glenoid)	222	73	193	78	0	0	1	1	0	0	0	0	0	0
Pectorals (coracoid)	120	47	89	65	0	0	0	0	1	1	0	0	0	0
Humeri	288	99	193	89	0	0	0	0	1	1	1	1	0	0
Ulnae	112	55	63	32	0	0	0	0	0	0	0	0	0	0
Radii	107	49	68	39	0	0	0	0	0	0	0	0	0	0
Metacarpals	3	3	0	0	0	0	0	0	0	0	0	0	0	0
Ilia	100	46	85	43	0	0	0	0	0	0	0	0	0	0
Ischia	70	36	74	46	0	0	0	0	0	0	0	0	0	0
Pubes	118	70	89	55	0	0	1	1	1	1	0	0	0	0
Pelves unspecified	0	0	15	0	0	0	0	0	0	0	0	0	0	0
Femora	212	90	115	69	1	1	0	0	0	0	0	0	1	1
Tibiae	79	27	39	19	0	0	0	0	0	0	0	0	0	0
Fibulae	58	24	33	18	0	0	0	0	0	0	0	0	0	0
Astragalocalcanei	6	6	0	0	0	0	0	0	0	0	0	0	0	0
Carpals/Tarsals	87	71	91	84	0	0	0	0	0	0	0	0	0	0
Phalanges (first and second)	73	58	89	81	0	0	0	0	0	0	0	0	0	0
Phalanges (third)	101	69	108	97	0	0	0	0	0	0	0	0	0	0
Humeri/Femora	14	0	12	0	0	0	0	0	0	0	0	0	0	0
Unspecified long bones	3	0	0	0	0	0	0	0	0	0	0	0	0	0
Total	1915	876	1672	954	1	1	2	2	5	5	1	1	1	1

Table S10: Tortoise butchery and cooking evidence from Lapita sites in the Southwest Pacific, percentages of tortoise skeletal element NISP with fresh fractures, cut marks, and burning.

Skeletal elements	Teouma			Vao		
	Fresh fracture	cut mark	burn t	Fresh fracture	cut mark	burn t
Maxillae	0	0	0	2.86	0	25.7 1
Mandibles	0	0	22.2 2	6.52	0	15.2 2
Unidentified tooth row fragment	0	0	0	0	0	0
Hyoids	0	0	0	0	0	0
Cervical vertebra	0	0	0	0	0	8.4
Thoracic vertebra	0	0	0	0	0	9.09
Caudal vertebra	0	0	0	0	0	8.7
Vertebra	0	0	0	0	0	12.5
Dermal shell fragments	0	0	4.76	0	0	0
Ribs	0	0	0	0	50	0
Pectorals (dorsal, acromion, glenoid)	1.35	5.41	1.35	4.66	1.55	30.5 7
Pectorals (coracoid)	3.33	1.67	1.67	2.25	0	17.9 8
Humeri	1.74	3.82	3.13	3.63	0.52	30.5 7
Ulnae	6.25	0	1.79	6.35	3.18	23.8 1
Radii	1.87	1.87	2.8	7.35	0	14.7 1
Metacarpals	0	0	0	0	0	0
Ilia	3	11	1	0	1.18	17.6 5
Ischia	2.86	2.86	1.43	6.76	1.35	33.7 8
Pubes	2.54	13.56	3.39	1.12	2.25	21.3 5
Pelves unspecified	0	0	0	0	0	0
Femora	1.42	4.72	1.42	0.87	1.74	15.6 5
Tibiae	0	3.8	2.53	7.69	0	30.7 7
Fibulae	0	1.72	3.45	3.03	0	15.1 5
Astragalocalcanei	0	0	0	0	0	0
Carpals/Tarsals	0	0	2.3	0	1.1	2.2
Phalanges (first and second)	0	0	2.74	0	0	2.25
Phalanges (third)	0	0	1.98	0	0	6.48
Humeri/Femora	0	0	0	0	0	0
Unspecified long bones	0	0	0	0	0	0

Table S11: Morphometric summary statistics for Teouma tortoise skeletal elements.

Element/portion	Number	Mean	Median	S.D.	Min	Max
Coracoid gl	5	57.3	62.4	17.2	37.5	78.4
Coracoid pr	51	21.7	22.4	6.5	7.7	33.7
Coracoid ds	3	38.8	41.8	12.5	25.1	49.5
Humerus gl	15	103.8	98.4	31.8	68	193.7
Humerus pr	32	37.8	38	9.6	17.9	55.6
Humerus ds	9	39.2	36.8	14.2	24.6	69.4
Radius gl	24	59.9	60.8	21	9.6	116.9
Radius pr	36	18.9	18.9	5.2	8.3	36.6
Radius ds	38	20.4	19.5	5.5	9.2	35.3
Ulna gl	26	70.1	68.4	18	35.7	104.6
Ulna pr	39	25.6	25.5	7.7	11.1	45.6
Ulna ds	38	25.9	25.7	7.9	11.8	47.2
Femur gl	15	95.6	104.5	24.3	42	122.2
Femur pr	33	36.5	39.1	11.5	12.1	60.9
Femur ds	9	37.8	40.9	9.7	23.3	48.5
Tibia gl	5	90.2	96.1	13.8	75	103
Tibia pr	11	27.9	29	5.5	17.8	34
Tibia ds	12	20.6	20	3.2	15.9	27.4
Fibula gl	5	69.7	75.2	23.8	37.6	95.3
Fibula pr	10	15.3	15.8	5.9	7.7	23.9
Fibula ds	13	12.2	12.9	4.3	5.4	20.3

Table S12: Morphometric summary statistics for Vao tortoise skeletal elements.

Element/portion	Number	Mean	Median	S.D.	Min	Max
Coracoid gl	1	76.3	76.3	0	76.3	76.3
Coracoid pr	44	21.4	20.7	6.5	8	40.6
Coracoid ds	0	0	0	0	0	0
Humerus gl	7	110.7	102	20.9	87.9	142.3
Humerus pr	20	39.7	39.9	9.3	16.3	57.8
Humerus ds	9	43.4	40.7	7.7	31.4	55.8
Radius gl	4	75	76	5.9	68.1	80.1
Radius pr	23	18.4	18.8	4.5	8.9	25.6
Radius ds	14	19.9	20.3	3.3	14.5	24.6
Ulna gl	10	64.2	67.1	12	44.4	82.5
Ulna pr	16	22.9	23.4	4.3	16.3	30.5
Ulna ds	20	19.6	20.5	4.9	11.5	27.9
Femur gl	8	105.3	101.3	19.7	84.8	142.6
Femur pr	25	38.3	37	7.8	25.4	56.5
Femur ds	6	45.2	47	10.2	33	57.4
Tibia gl	1	114.1	114.1	0	114.1	114.1
Tibia pr	2	30.9	30.9	8.4	25	36.8
Tibia ds	7	20.5	19.6	3.5	16.3	26.9
Fibula gl	1	83.8	83.8	0	83.8	83.8
Fibula pr	3	14.1	15.6	4.7	8.8	17.8
Fibula ds	4	13.9	13.9	0.8	13	14.8

Table S13: Database codes for zooarchaeological analyses.

Table S14: Isotopic values for the Yanuca femur specimen.

Site	Sample ID	d13c v PDB	d15N v AIR
Yanuca	16/81/2/3/4/1	-24.8	9.5

Supplementary Text

Teouma, Efate Island, Central Vanuatu

The Teouma Lapita cemetery/settlement site is located at Teouma Bay situated on an ancient upraised beach platform on the south coast of Efate Island, central Vanuatu (*1*). In total, 473 m^2 in the main cemetery/settlement area were excavated in 6 field seasons (2004-2006, 2008-2010). The excavations sampled a distinctive Lapita cemetery and an adjacent midden refuse deposit contemporary with the use of the cemetery. These early deposits were laid down on a natural orange tephra deposit overlying the limestone reef beginning ca. 3000 BP. By ca. 2700 BP the cemetery was no longer in use and the deposition of a Late-Lapita midden (Layer 2) had expanded over the top of the cemetery (*2*). This midden became thinner and less concentrated with faunal material as it transitioned into Post-Lapita Arapus and Erueti type ceramics in the upper younger layers (*2*).

A total of 1915 tortoise bones (MNI = 68, total weight = 11878.7 grams) were identified from Teouma (Tables S2, S9), considerably more than the 405 bones of *?Meiolania damelipi* originally reported when the species was first described (*3*). They were concentrated within the basal cemetery (NISP= 161, 0.45/m^2) and adjacent Lapita settlement layers (NISP=292, 2.4/m^2) that intruded into the natural orange tephra, and in the later Lapita deposits (basal Layer 2, NISP=872, 2.5/m^2) immediately after the use of the cemetery by 2700 BP. Numbers dramatically declined to 0.07 tortoise bones per m^2 around the transition to the Post-Lapita period. Only a few bones were associated with the upper Post-Lapita midden and these are likely to have been specimens located at the boundary between the Lapita and Post-Lapita deposits, although minor post-depositional vertical movement of faunal material may also have contributed. Proximal limb bones including humeri, femora, pectorals (fused fragments including the acromion, glenoid, and the dorsal scapular process, and the unfused coracoid), as well as pelves (pubes) are quite frequent while smaller

quantities of more distal limb bones (radii, ulnae, carpals, metapodials, tibiae, fibulae, tarsals, and phalanges), skull bones, vertebra, ribs, and pelves (ischia, ilia), were identified. Small proportions of pectoral bones (coracoid, scapula based on each of the dorsal scapular process, acromion, and glenoid), humeri, ulnae, radii, femora, tibiae, fibulae, pelves (pubes, ilia, ischia) show signs of burning and butchery either with cut marks and/or butchery fractures (Table S10). Small proportions of burn marks were also observed on the skeletal extremities due to less meat being present on these body portions during either cooking or disposal (Table S10). These include on one mandibular fragment, small numbers of carpals/tarsals, phalanges and a dermal shell fragment. The low proportions of burning on the skeletal material may indicate that steam cooking in earth ovens was the preferred method.

Vao, Northern Vanuatu

The Lapita site on the small island of Vao (<2 km²), located off the northeast coast of Malakula, northern Vanuatu, is situated on a small uplifted sheltered beach terrace facing Malakula. Three field seasons between the years 2002-2004 were conducted during which 36 m² of the estimated 4000 m² site area were excavated (*4*). The site has five main prehistoric stratigraphic layers (*4*). Layer 1 is a rich dark brown soft tephra-rich layer comprising volcanic ash from irregular falls and anthropogenic-derived debris accumulated since ca. 2000 BP which wholly underlies historic village houses and associated coral cobble compounds with few faunal remains. Layer 2 consists of a dense layer of coral gravel interpreted as formerly associated with prehistoric household compounds dated to ca. 2300-2000 BP and including Post-Lapita plain-ware and moderately abundant faunal material. Layer 3 is a dark brown sandy cultural layer dated to the Late Lapita period based on associated ceramics, which include dentate stamp sherds, and has abundant faunal remains. Layer 4 is a dark brown sandy silt with the greatest concentration of cultural material (ceramics, faunal remains, shell ornaments, stone tools). It is the earliest Lapita component of the site, radiocarbon dated between

3000-2600 cal BP (*4*). Underlying the early Lapita midden is the natural beach sand (Layer 5) which had cultural material compressed and mixed into the uppermost 20-50 centimetres.

Tortoise bones are far more abundant per meter square at Vao compared to Teouma. In total 1672 (Tables S3, S9) *?M. damelipi* type tortoise bones (46/m^2) were recovered from the Vao excavations representing an MNI of 49 (total weight = 7614.1 grams) of which the greatest concentration was in Layer 4 (NISP=1001, 27.8/m^2), but they were also ubiquitous in Layer 5 (NISP=570, 15.8/m^2) with notably fewer vertebrate remains in the Late Lapita Layer 3 (NISP=38, 1.05/m^2). Four tortoise bones (0.11/m^2) are associated with the Post-Lapita deposits of Layer 2, but these were most likely disturbed from below or were excavated at the boundary of the Lapita deposits. The Vao tortoise assemblage is characterised by high proportions of burnt bone suggesting differences in cooking and tortoise utilisation compared to Teouma, although evidence of butchery (cut marks and smooth regular fractures) per skeletal element were proportionately low, much like they were at Teouma (Table S10). This quantity of tortoise bone from this single site located on such a small island again infers that people were travelling to hunt tortoises. The big island of Malakula, visible across a channel from Vao, would have provided the ideal hunting ground.

Uripiv, Northern Vanuatu

The Velavi Lapita site is situated on Uripiv island, another small island <2 km^2 off the northeast coast of Malakula in Northern Vanuatu. Like Vao, the site is on the uplifted beach terrace (ca. 7 m above sea level and 50 m inland) on the sheltered side of the islet facing westwards towards Malakula. To date 75 m^2 of the estimated 3000 m^2 site have now been excavated during five field seasons (2001-2002 and 2009-2011) (*4*). The site, while sharing a remarkably similar chronostratigraphy with Vao, is a slightly later Lapita deposit based on form and style of the contained ceramics. A single tortoise femur (Fig. S2, Table S5, #SCH 250 collected 18/08/2001) was recovered and identified from the sterile beach sand deposit of Layer 5 immediately underlying the Lapita midden in Test Pit 8. The femur surface was largely encrusted with calcareous beach sand and was heavily gnawed by rats

indicating that it was on the beach surface after Lapita people arrived with rats about 3000 BP. That it was gnawed means it was likely fresh bone and so attractive to rats rather than a fossil. We therefore consider it was probably deposited coeval with the first people occupying the site and is likely to have been buried within the upper beach sands by trampling before a discrete midden then buried it further.

Arapus, Efate Island, Central Vanuatu

The Arapus site (immediately Post-Lapita based on ceramic form and style) is located adjacent to the Mangaasi site on the northwest leeward coast of Efate Island in central Vanuatu (*5*). The Neolithic settlers established themselves adjacent to the shore parallel with the beach, as did later generations. Due to tectonic uplift over 1500 years, however, the beach side settlements continued to shift and the earliest component of the site, the Arapus Phase, is now situated some 125-150m from the shore. A total of 53, 1 x 1m test pits (53 m^2) and three separate larger areas comprising $4m^2$, $9m^2$ and $4m^2$ ($17m^2$) were excavated between 1999 and 2001-2003. The early Arapus Phase of the site is dated to around 2800 BP (range 3197-2712 cal BP) (*5*). It is the earliest human settlement found so far on the ecologically less diverse northwest side of Efate. Two *?M. damelipi* (Table S6) type tortoise bones were identified from basal cultural deposits overlying sterile beach sand. These include a large pectoral element (a near complete scapula with partial acromion and dorsal scapular process) (Fig. S3, #SCH 267, collected 22/06/1999) from TP 4 (135-150cm deep) and a pubis fragment (#SCH 311, collected 24/06/1999) from TP 28 (200-210cm depth). These bones were moderately weathered possibly from surface exposure and showed no obvious signs of butchery or cooking.

Port Olry, Santo Island, Northern Vanuatu

The Port Olry site is today situated about 1 km away from the current shoreline on the northeast coast of Santo in Northern Vanuatu. At the time of settlement the shoreline would have been

adjacent to the site, but as tectonic uplift occurred and sea level fell, the shoreline has prograded to its current position. The site is a Lapita settlement with peripheral immediately Post-Lapita deposits. Samples from excavations of the periphery in 2006 returned Post-Lapita dates ca. 2720-2350 cal BP (*6*) and were associated with immediately Post-Lapita ceramics without tortoise bones. Subsequent excavations in 2011 (10, 1x1m test pits), revealed the early Lapita area of the site with a Lapita deposit close to the surface. The Lapita deposit (ca. 60 cm deep) extends to the base of cultural deposition on top of the underlying sterile beach sand deposits. Five tortoise bones (MNI = 1; weight = 24.82 gm) were identified from sparse and fragmentary faunal material recovered throughout the 60 cm of this deposit which has yet to be radiocarbon dated. None of these showed signs of butchery or cooking.

Naigani, Viti Levu, Fiji

The VL 21/5 Lapita site on the small island of Naigani (<2 km^2) off the Northeast coast of Viti Levu in Fiji was first excavated in 1981 and again in 2000 (*7*), during which ca. 120 m^2 were excavated. The site is now approximately 100 m inland, as the shoreline has prograded since initial occupation of the site between 3270-2410 cal BP, when it was situated on a beach dune adjacent to the shore (*7*). A single humerus (Fig. S1, Tables S8-S9, #SCH 150, collected in 1981) indistinguishable from those of *?Meiolania damelipi* was identified from Square 14 on the southern edge of the site from Layer A3. The humerus has been extensively weathered and abraded since deposition, like the majority of bones recovered from the site, due to poor preservation conditions and gardening disturbance.

Yanuca, Fiji

The Yanuca Lapita site (VL 16/81) is located 11 km west of the Sigatoka River on the north coast of Yanuca Island, Fiji. The small island (0.48 km^2) is composed of limestone overlain by shallow sandy or sand-clay soils and is separated from Viti Levu by a 180 m wide tidal channel. Archaeological investigations began on the island in 1965 when Lawrence and Helen Birks excavated test pits in rock

overhangs on the northwest side of the island and made surface collections of pottery followed by additional test pit excavations by Hunt during the 1970s (*8*). The Lapita component of the site is thought to have been minor, as it was in a limestone pocket that was completely excavated during the 1960s, with the early deposits dated to between 2930 and 2730 cal BP (*8*). A total of 47 m^2 was excavated during the Birks and Hunt excavations. The Lapita material was associated with the basal 40-50 cm of cultural deposit (Zone 3) which overlay a culturally sterile natural sandy substrate (Layer E) (*8*). Very little faunal material was observed in the prehistoric layers and no screening was employed. The *?M. damelipi* type femur (Fig. S2, #16/81/2/3/4/1 collected 1965-66) was recovered ~165 cm below the excavation surface in this basal Zone 3 Lapita deposit. The fauna were analysed by Hunt who identified the tortoise femur as ?pig (*Sus scrofa*), and for many years the 'pig' bone was used to support the presence of Lapita horticulture in Remote Oceania (*9*). The femur was moderately weathered post-deposition and had been partially burnt before burial. It was probably from a tortoise that was cooked, with heating likely making the bone susceptible to post-depositional degradation. The isotopic d13C value of -24.8 relative to the VPDB standard PDB of this bone indicates a terrestrial plant diet similar to Teouma tortoises (-23.20, -22.78), although the d15N value of 9.5 per mil relative to AIR was much higher than the Teouma specimen (Table S14).

Systematic Paleontology

?Meiolania damelipi White et al. (*3*).

Holotype AMF136641, right humerus, Teouma, Efate Island, Vanuatu, 2006.

Comments.

The long bones of *?Meiolania damelipi* were described by White et al (*3*) as more gracile compared to *Meiolania platyceps* whose long bones are stocky and thick by comparison (*10*). Features of the humerus, including proximal articulating surface and medial and lateral processes, were used by White et al. (*3*) to diagnose the species, as cranial material of *?M. damelipi* was not available at the

time for comparison. The referral of the present specimens to *?Meiolania damelipi* is based on their similarity to the type specimens and to associated topotype material from Teouma. Here we provide osteological details in support of the present referral of specimens to *?Meiolania damelipi* for the skeletal elements humeri, femora, and scapulae: acromion, glenoid, dorsal scapular process, from the seven Lapita sites from Vanuatu-Fiji discussed herein (Figs. S1-S3). Summary statistics of measurements from tortoise long bones from Vao and Teouma can be found in Tables S11-S12.

Humeri were described by White et al. (*3*) as having less expanded ends compared to *Meiolania platyceps*, estimated lengths ranging from 40-140 mm, a hemispherical proximal articular surface offset dorsally from the shaft, and differed from M*. platyceps* and *M. mackayi* with a less expanded lateral process, a more proximally projecting medial process, and the ectepicondylar foramen was preceded by a wider groove on the dorsal facies of the shaft. Here we record some additional, distinctive morphological traits (Fig. S1) not found in *M. platyceps* that are shared by topotype specimens from Teouma (e.g., #SCH: 589, 9/07/2008, Area 3a3.5 Layer 2 spit 4, humerus left), and those from Vao (#SCH: 251, 7/08/2003, TP 4, layer 4 130-140cm, humerus left), Port Olry (#SCH: 14, 18/06/2011, Area 1 TP 9, depth 40-60cm, humerus left) and Naigani (#SCH: 150, 1981, Square 14, Layer A3, humerus left). These include a small ventral proximal fossa, which is situated near where the proximal shaft meets the intertubercular fossa, and a second fossa on the dorsal proximal shaft. These fossae vary in size and depth with some Teouma and Vao specimen being more pronounced than others, but are always present. The ventral proximal fossa are absent in the *M. platyceps* humeri specimens (AM F49141, AM F18748), while the dorsal proximal fossa is much less pronounced and has a shallow v shape compared to the deep oval shaped dorsal proximal fossae of *?M. damelipi*. In addition, humeri of ?*M. damelipi* consistently show distinctive muscle attachment scars on the anterior proximal shaft and the posterior proximal shaft in all specimens. These consistently appear as elongated patterns of fibrous grooves with crenulations along the proximal shaft. In *M. platyceps* (AM F 49141, AM F18748) the anterior proximal muscle scar differs from *?M. damelipi*. In the latter, it appears as a rectangular groove with crenulations inside and extends up to

the proximal lateral process. In the former, it takes a different form as a pronounced narrow ridge protrusion with no groove extending from the distal portion of the dorsal proximal shaft fossa up to the lateral process. The posterior proximal shaft muscle attachment scar is absent from some ?*M. damelipi* specimens, but when it is present it appears as a narrow long shallow groove extending diagonally from the medial process to a point approximately one third of the shaft. In some specimens, the groove is shorter and less perceptible. In *M. platyceps*, it is also not always present (absent in AM F49141), but when it does appear it is a shallow groove extending straight down the shaft from the medial process (AM F18748).

Complete humeri have a greatest length ranging from 68 to 194 mm at Vao and Teouma (Tables S11-S12) compared with 125 mm (AMF:18742) to 205 mm (AMF:16850) for *Meiolania platyceps* from Lord Howe Island (*10*) making them comparable in absolute maximum length, although those of *M. platyceps* are far stouter. The average maximum length is 103.8 mm for Teouma and 110.7 mm for Vao. Maximum proximal width ranges between 16.3 to 57.7 mm (mean; Teouma = 37.7 mm, Vao = 39.7 mm) and maximum distal width range is 24.6 to 69.4 mm (Mean; Teouma = 39.2 mm, Vao = 43.4 mm). They combine to give average proportions (mean distal humeri width/mean humeri length; Teouma = 0.38, Vao = 0.39) that indicate that the humeri from Teouma and Vao are morphometrically similar. The high standard deviations indicate a wide variability in size of tortoise humeri at Teouma and Vao, reflecting hunting of all age classes.

Femora are described by White et al. (*3*) as stocky ranging from 45-145 mm in shaft length with a large, wider than long, hemispherical head that is directed more dorsally to the shaft in comparison to *M. platyceps* and that does not project proximally past the trochanter major. Unlike *M. platyceps* the major and minor trochanter have a similar proximal extent and are interconnected ventrally by a bony web that encloses a deep intertrochanteric fossa. Here (Fig. S2) we identify two additional distinguishing femoral features using topotype specimens from Teouma (e.g., #AW: 404, 21/06/2006, 3B1.5, Layer 3, right femur), and also observed in Vao (e.g., #SCH: 54, 29/10/2004,

TP14, layer 5, 180-190 cm depth, right femur), Uripiv (#SCH: 250, 18/08/2001, TP8, Layer 5, 220-240 cm depth, right femur) and Yanuca (#16/81/2/3/4/1, 1965-66, Trench 2, zone 3 spit 4, 165 cm depth, right femur) specimens. Located on the posterior proximal shaft there is a distinct fossa for a muscle attachment, and on the anterior proximal shaft there is a series of fossae and grooves for muscle attachments (Fig. S2). For *?M. damelipi* the posterior proximal shaft fossa appears as a long deep narrow crenulated depression that extends from the trochanter major straight down the edge of the shaft to approximately 1/3 of the shaft length. The posterior proximal fossa for *M. platyceps* appears as a shallow crenulated depression that encompasses the entire anterior proximal portion of the upper shaft with a ridge in the centre for AM F10773 but is barely perceptible for AM F18759. The anterior proximal shaft fossa for *?M. damelipi* is a moderate crenulated depression present in all specimens, although it varies from specimen to specimen in how pronounced it is. This fossa is absent from *M. platyceps* specimens (AM F10773, AM F18759).

M. platyceps femora vary greatly in size, the longest being AMF: 1203 at 213 mm, but the incomplete AMF: 1788 is estimated to be as large as 274 mm (*10*). Femora of *?M. damelipi* are smaller (Table S11) and have a maximum complete length ranging between 42 mm to 122 mm (Mean = 95.6 mm) for Teouma specimens and 84.8 mm to 142.6 mm (mean = 105.3 mm) for Vao specimens (Table S12), again reflecting the wide range of age classes hunted. Proximal maximum width is similarly variable for Vao (mean = 38.3 mm) and Teouma (mean = 36.5 mm), ranging between 12.1 mm to 60.9 mm while distal femora width for both sites ranges between 23.3 mm to 57.4 mm (Teouma mean = 37.8 mm; Vao mean = 45.2). Proportionate data (mean proximal humeri width/mean humeri length and mean distal humeri width/mean humeri length) also indicate similarities between Teouma (pw/l = 0.38, dw/l = 0.4) and Vao (pw/l = 0.36, dw/l = 0.43).

The shoulder girdle is triradiate i.e., it has three radiating branches from the glenoid, the dorsal scapular process and the acromion of the scapula, and the coracoid. The scapulae of *?M. damelipi* are characterised by the glenoid having no supporting neck, and the dorsal scapular process and the

acromion diverging at approximately 105°, compared to 120° for the deep shelled *M. platyceps* (*3, 10*); suggesting that the Vanuatu tortoises had less deep shells by comparison. Another difference from *M. platyceps* is that the coracoid remains unfused from the scapular glenoid and is more elongate, which indicates *?M. damelipi* had a lower body profile (*3*). Here (Fig. S3) we identify another distinctive feature for *?M. damelipi*, a muscle attachment scar on the lateral acromion surface. This attachment scar appears as a projecting crenulated ridge which often extends from the lateral surface of the acromion to the articulation at the glenoid for the coracoid, although there is some variation in how pronounced this ridge is. In *M. platyceps* it is not present in specimen AM F18727 and a smooth surface in this region was observed.

References

1. Bedford, S. *et al*. A cemetery of first settlement: Teouma, South Efate, Vanuatu/ Un cimetière de premier peuplement: le site de Teouma, sud d'Efaté, Vanuatu in *Ancestors/Lapita: Ancétres Océaniens.* (eds. Sand, C., Bedford, S.) 140-161 (Musée du Quai Branly/Somogy, 2010).

2. Petchey, F., Spriggs, M., Bedford, S., Valentin, F. The chronology of occupation at Teouma, Vanuatu. *Journal of Archaeological Science Reports.* **4**, 95-105 (2015).

3. White, A., Worthy, T., Hawkins, S., Bedford, S., Spriggs, M. Megafaunal meiolaniid horned turtles survived until early human settlement in Vanuatu, Southwest Pacific. *PNAS*. **107**(35), 15512-15516 (2010).

4. Bedford, S., Buckley, H., Valentin, F., Tayles, N., Longga, N. Lapita Burials, a New Lapita Cemetery and Post-Lapita Burials from Malakula, Northern Vanuatu, Southwest Pacific. *Journal of Pacific Archaeology.* **2**(2), 26-48 (2011).

5. Bedford, S., & Spriggs, M. Crossing the Pwanmwou: Preliminary Report on recent excavations adjacent to and southwest of Mangaasi, Efate Vanuatu. *Archaeology in Oceania.* **35**, 120-126 (2000).

6. Bedford, S., & Spriggs, M. Northern Vanuatu as a Pacific Crossroads: the Archaeology of Discovery, Interaction, and the Emergence of the "Ethnographic Present”. *Asian Perspectives.* **47**(1), 95-120 (2008).

7. Irwin, G. *et al*. Further investigations at the Naigani Lapita site (VL 21/5), Fiji: excavation, radiocarbon dating and palaeofaunal extinction. *Journal of Pacific Archaeology.* **2**(2), 66-78 (2011).

8. Clark, G., Anderson, A. The Age of the Yanuca Lapita Site, Viti Levu, Fiji. *New Zealand Journal of Archaeology.* **22**(2000), 15-30 (2001).

9. Worthy, T, & Clark, G. Bird, mammal and reptile remains in *The early prehistory of Fiji* (eds. Anderson, A., Clark, G.) 231-258 (Terra Australis, 2009).

10. Gaffney, E.S. The postcranial morphology of *Meiolania platyceps* and a review of the Meiolaniidae. *Bulletin of the American Museum of Natural History*. **229**, 1-166 (1996).
